# Protective Effects of HDL Against Ischemia/Reperfusion Injury

**DOI:** 10.3389/fphar.2016.00002

**Published:** 2016-01-25

**Authors:** Monica Gomaraschi, Laura Calabresi, Guido Franceschini

**Affiliations:** Centro E. Grossi Paoletti, Dipartimento di Scienze Farmacologiche e Biomolecolari, Università degli Studi di MilanoMilan, Italy

**Keywords:** high density lipoproteins, HDL cholesterol, synthetic HDL, ischemia–reperfusion injury, acute coronary syndrome

## Abstract

Several lines of evidence suggest that, besides being a strong independent predictor of the occurrence of primary coronary events, a low plasma high density lipoprotein (HDL) cholesterol level is also associated with short- and long-term unfavorable prognosis in patients, who have recovered from a myocardial infarction, suggesting a direct detrimental effect of low HDL on post-ischemic myocardial function. Experiments performed in *ex vivo* and *in vivo* models of myocardial ischemia/reperfusion (I/R) injury have clearly shown that HDL are able to preserve cardiac function when given before ischemia or at reperfusion; the protective effects of HDL against I/R injury have been also confirmed in other tissues and organs, as brain and hind limb. HDL were shown to act on coronary endothelial cells, by limiting the increase of endothelium permeability and promoting vasodilation and neoangiogenesis, on white blood cells, by reducing their infiltration into the ischemic tissue and the release of pro-inflammatory and matrix-degrading molecules, and on cardiomyocytes, by preventing the activation of the apoptotic cascade. Synthetic HDL retains the cardioprotective activity of plasma-derived HDL and may become a useful adjunctive therapy to improve clinical outcomes in patients with acute coronary syndromes or undergoing coronary procedures.

Besides being independent predictors of the incidence of acute ischemic diseases, low plasma levels of HDL-C were shown to be associated to an unfavorable prognosis; indeed, plasma levels of HDL-C at admission are inversely associated with the severity of the ischemic insult, with 1-year mortality, and with the recurrence of subsequent clinical events (Olsson et al., 2005; Ghazzal et al., 2009; Yeh et al., 2013). The relationship between HDL-C and prognosis is not likely due to an accelerated atherogenesis, but may suggest a detrimental effects of low HDL on post-ischemic recovery; low levels of HDL-C are indeed associated with post-ischemic left ventricular dysfunction independent of coronary atherosclerosis severity (Kempen et al., 1987; Wang et al., 1998, 1999). Thus, in recent years several studies have been devoted to assess whether plasma-derived and synthetic HDL (sHDL) could reduce ischemic injury using *in vitro*, *ex vivo*, and *in vivo* models, and to investigate the HDL components and the mechanisms responsible for the protective effects.

## HDL and Myocardial I/R Injury

Acute myocardial infarction is the result of the occlusion of an epicardial coronary artery, generally due to the sudden rupture of an atherosclerotic plaque, limiting the perfusion of the cardiac region distal to the occlusion site (the so-called “area at risk”). If a timely reperfusion is not achieved and no collateral circulation is present, cardiomyocytes will be irreversibly damaged and substituted by fibrous tissue with impairment of contractile function. Thus, reperfusion is mandatory to limit infarct size and improving post-ischemic cardiac function; however, reperfusion itself is associated with additional myocardial damage, generating the definition of “ischemia/reperfusion (I/R) injury” (Carden and Granger, 2000).

To evaluate the I/R pathology and possible therapeutic approaches to limit I/R damage, different models can be used, ranging from *ex vivo* isolated hearts to *in vivo* ligation of coronary arteries in small to large mammals. Isolated hearts are useful for their easiness of manipulation (i.e., duration and nature of I/R) and allow a complete biochemical, physiological, and morphological evaluation. However, the isolated heart has a limited life-span and does not allow to evaluate the role of hemodynamic and systemic factors, as *in vivo* models do (i.e., permanent or temporary ligation of the left descending coronary artery, LAD; Hearse and Sutherland, 2000). Small animals as rodents are of great help for their ease of breeding, low cost, and availability of transgenic models, but their translational relevance is limited by the huge anatomical and physiological differences with humans; large animals as pigs, dogs, and primates surely display a much closer phenotype to humans and represent a mandatory step toward clinical trials, but still suffer of differences in the extent of collateral blood flow and resistance to infarct development (Ibanez et al., 2015).

Plasma-derived HDL were first shown to limit I/R injury in an *ex vivo* model on isolated rat hearts retroperfused through the aorta according to the Langendorff technique (Calabresi et al., 2003b). A moderate I/R injury was induced by reducing the perfusion flow for 20 min and then reperfusing the hearts for additional 30 min; this protocol resulted in an remarkable post-ischemic impairment of contractile function, increase of coronary resistance to perfusion flow and cardiac necrosis. In this model, HDL significantly limited I/R injury, when given immediately before ischemia or during the first minutes of reperfusion; the ability of HDL to limit I/R injury even when administered after ischemia could be clinically relevant and was further confirmed in isolated mice hearts treated with HDL at reperfusion (Frias et al., 2013).

Cardioprotection by HDL against I/R injury was definitely proven *in vivo*. HDL were given to mice 30 min before the ligation of left descending coronary artery; after 30 min, blood flow was restored for additional 24 h. Mice treated with HDL displayed a large reduction of infarct size with less myocyte necrosis and inflammation compared to vehicle-treated animals (Theilmeier et al., 2006). In agreement with this evidence, mice lacking apoA-I: display increased infarct size after LAD in a gene-dose-dependent way (Dadabayev et al., 2014).

## sHDL and Myocardial I/R Injury

The translational potential of HDL as cardioprotective agents against I/R injury is hampered by several difficulties, including their heterogeneity and safety concerns; these limitations can be overcome by the use of sHDL. These particles are usually composed by recombinant proteins as apoA-I, the main protein component of HDL, and phospholipids, generally phosphatidylcholine, and are now undergoing the clinical phase of development as plaque-stabilizers in the context of an acute coronary syndrome (ACS; Krause and Remaley, 2013). By using the same *ex vivo* experimental protocol described above, we showed that these sHDL are able to limit I/R injury when given before or after the ischemic insult and that the two sHDL components, apoA-I and phospholipids, are not effective when given alone (Rossoni et al., 2004). However, the effect of sHDL is about 50% lower than that of plasma-derived particles, suggesting that the basic sHDL composition should be modified in order to achieve maximal cardioprotective activity. Marchesi et al. (2004) confirmed the sHDL-mediated cardioprotection against I/R injury *in vivo* by testing the administration of sHDL containing the apoA-I Milano variant to rabbits before temporary LAD; at reperfusion infarct size was reduced in sHDL-treated compared to vehicle-treated animals. Several agents are able to inhibit infarct size in animal models, however, a parallel improvement of cardiac function has not been always observed. HDL were instead shown to limit the decline of contractile function and the increase of coronary resistance to perfusion flow; in addition, sHDL are able to reduce the duration of ventricular tachycardia or fibrillation at reperfusion when given to rats before LAD (Imaizumi et al., 2008). Contrary to what observed *ex vivo*, where apoA-I had to be associated with phospholipids to exert cardioprotection, infarct size after LAD was significantly reduced in rats infused with unlipidated apoA-I 10 min before artery ligation (Hibert et al., 2013); this discrepancy is likely explained by the rapid interaction of infused apoA-I with circulating lipoproteins and the increase of endogenous HDL pool, which is not possible in blood-free experimental settings.

As stated above, an optimization of sHDL composition is needed to achieve a cardioprotective effect comparable to that of plasma-derived lipoproteins. Changes could involve the protein and/or the lipid components, and the inclusion of proteins that circulate in the bloodstream bound to HDL. For what concerns the protein composition, apoA-I can be substituted by apoA-I mimetic peptides, which surely represents an advantage for the large-scale preparation of sHDL, or apoA-I variants. In the *ex vivo* model of I/R injury, particles containing 37 pA displayed the same cardioprotective activity of those with apoA-I (Gomaraschi et al., 2008), while sHDL containing the apoA-I Milano variant display a superior protection against I/R injury in terms of reduction of cardiomyocyte damage (Calabresi et al., 2006; Marchesi et al., 2008). Regarding the lipid composition, a key indication comes from the study by Theilmeier et al. (2006), in which the cardioprotective effect of plasma-derived HDL was shown to be mediated by the interaction of the carried sphingolipid sphingosine-1-phosphate (S1P) with its G-protein coupled receptor. The addition of S1P to sHDL containing apoA-I and phosphatidylcholine resulted in a significant increase of sHDL-mediated cardioprotection *ex vivo* and *in vivo* (Brulhart-Meynet et al., 2015).

## HDL and I/R Injury in Other Tissues

Some evidences suggest that the protective role of HDL against I/R injury is not limited to the cardiac muscle but could be extended to other organs and tissues. In particular, recent studies demonstrated that HDL can limit I/R injury also in brain, hindlimb, kidney, intestine, liver, and lung. Both plasma-derived and sHDL were tested in stroke models. In the first study, cerebral ischemia was induced in rats by temporary ligation of the middle cerebral artery (MCA) for 30 min and apoA-I containing sHDL were infused before ischemia; a dramatic reduction of brain necrotic area was observed in animals treated with sHDL compared to vehicle-infused ones 24 h after MCA reopening (Paterno et al., 2004). The effect of plasma-derived HDL was tested in a model of focal cerebral ischemia induced by embolization of a preformed clot in the MCA; in this case, HDL were administered immediately after stroke onset and caused marked reductions of mortality rate, infarct volume, blood–brain barrier breakdown, and neurological deficits at 24 h (Lapergue et al., 2010). Interestingly, HDL exerted their protective effects even when infused up to 5 h after stroke onset. Using the same experimental model HDL were also shown to decrease the rate of mortality and hemorrhagic transformation induced by tissue plasminogen activator (Lapergue et al., 2013). The protective effects of sHDL and unlipidated apoA-I were assessed in the context of hindlimb ischemia. Unilateral hindlimb ischemia was induced by resecting the right femoral and saphenous artery and apoA-I containing sHDL were injected twice per week, starting 1 week before surgery; 4 weeks after surgery, animals treated with sHDL displayed superior blood flow recovery and capillary density than vehicle-treated animals (Sumi et al., 2007). Similarly, the infusion of apoA-I every second day after ligation of the femoral artery increased neovessel formation and blood perfusion in mice (Prosser et al., 2014). HDL markedly reduced renal dysfunction and I/R injury when given before arterial and venous clamping: at reperfusion serum, urinary and histological markers confirmed less renal dysfunction and cellular damage in HDL-treated rats (Thiemermann et al., 2003). Intestinal I/R injury was also attenuated by sHDL: when given before splanchnic artery occlusion, sHDL limited histological and clinical signs of ileum injury and the infiltration of inflammatory cells at reperfusion (Cuzzocrea et al., 2004). The overall protective effect of both plasma-derived and sHDL against I/R injury is well recapitulated in a model of multiple organ dysfunction syndrome; this syndrome is a typical consequence of hemorrhagic shock in which I/R injury occurs at several organs due to a reduced blood flow and oxygen supply. A rapid fall of arterial pressure was obtained by blood withdrawn from rat carotid artery and subsequent resuscitation by reinjection of blood and isotonic saline (Cockerill et al., 2001b). The administration of HDL or sHDL immediately before resuscitation reduced I/R damage in the liver, kidneys, pancreas, brain, muscles, lung, and intestine, as demonstrated by serum markers of cell necrosis (i.e., transaminases, creatinine, creatinine kinase, etc.) and histological analyses: organs from HDL-treated rats showed less edema and loss of normal tissue structure, less cellular infiltration and endothelial/epithelial expression of cell adhesion molecules (CAMs; Cockerill et al., 2001b).

## Mechanisms of HDL Protection Against I/R Injury

**FIGURE 1:**
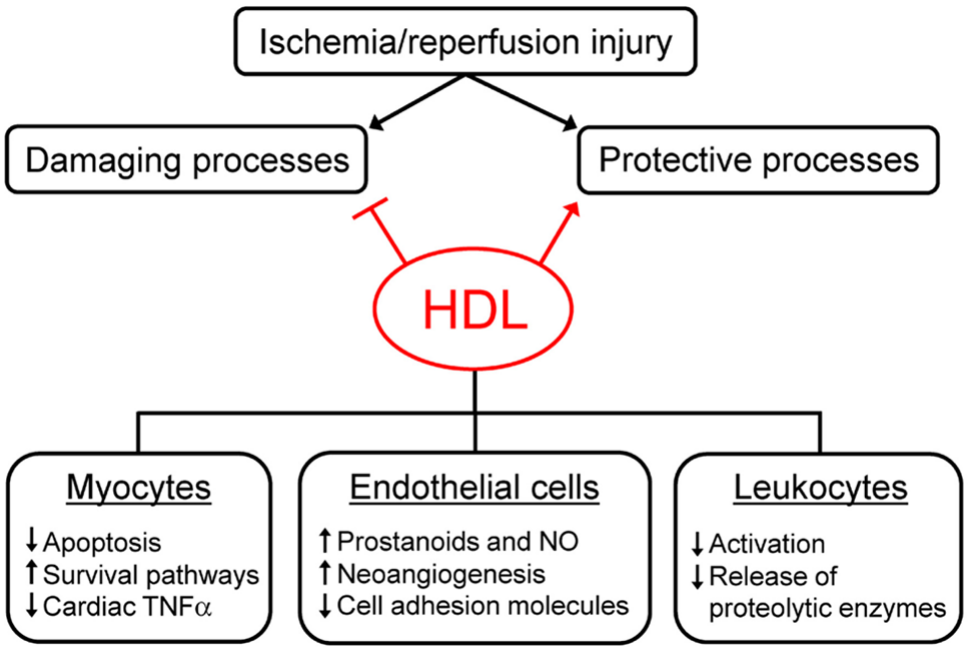
**Mechanisms of high density lipoprotein (HDL)-mediated protection against ischemia/reperfusion injury.** HDL can reduce ischemia/reperfusion (I/R) injury by inhibiting damaging processes and by promoting protective responses elicited by tissues after ischemia/reperfusion. HDL exert their effect on different cell types, as cardiomyocytes, endothelial cells, and leukocytes. See text for details.

In recent years, several studies have been performed to elucidate the mechanisms underlying plasma-derived and sHDL-mediated protection against I/R injury. The proposed mechanisms can be divided into two groups: the stimulation of endogenous protective responses and the inhibition of damaging processes (**Figure 1**). Protective responses are aimed at sustaining cell viability and at ensuring the maximal perfusion of ischemic tissues. HDL can promote the activation of different pro-survival pathways, as the survivor activating factor enhancement pathway (SAFE; Frias et al., 2013). In addition, HDL administration causes the release of molecules favoring vasodilation and myocyte relaxation as prostanoids and nitric oxide *ex vivo* and *in vivo* (Calabresi et al., 2003b; Theilmeier et al., 2006). The increase of HDL-mediated NO production through the PI3K/Akt pathway is almost blunted in the absence of the S1P3 receptor and is involved in the anti-arrhythmogenic effect of HDL (Theilmeier et al., 2006; Imaizumi et al., 2008).

Damaging responses are generally consequent to the activation of the inflammatory cascade, the infiltration of circulating leukocytes and the increased oxidative stress. HDL can limit the alterations of endothelial barrier permeability, the extravasation of circulating cells and the release of pro-inflammatory and pro-oxidant molecules like cytokines, chemokines, and reactive oxygen species. In particular, HDL modulate cardiac TNFα content, which seems crucial in the early phase of I/R injury, since it combines the capacity to directly affect myocyte function with the ability to trigger an inflammatory response (Frangogiannis et al., 1998; Meldrum, 1998). Preformed TNFα can be immediately released in the ischemic tissue by the degranulation of resident mast cells and the cleavage of membrane-bound TNFα (Frangogiannis et al., 1998; Kupatt et al., 1999), causing a significant increase of TNFα levels in ischemic hearts (Calabresi et al., 2003b). Both plasma-derived and sHDL act as TNFα scavengers, able to bind TNFα and to remove it from the ischemic tissue, thus preventing its damaging effects (Calabresi et al., 2003b; Rossoni et al., 2004). The mechanisms outlined above suggest that HDL-mediated protection against I/R injury is due to their ability to act on myocytes, but also on endothelial cells and infiltrating leukocytes, as confirmed in cell studies mimicking I/R injury through hypoxia-reoxygenation and glucose deprivation protocols (**Figure 1**).

### Cardiomyocytes

High density lipoprotein limits cardiomyocyte apoptosis in the ischemic area following LAD (Theilmeier et al., 2006). These findings were confirmed *in vitro* on cardiomyocytes under glucose deprivation or subjected to hypoxia and are linked to the inhibition of the mitochondrial permeability transition pore opening consequent to the activation of the SAFE pathway (Theilmeier et al., 2006; Frias et al., 2013). The preservation of myocyte viability after hypoxia-reoxygenation is mediated by S1P through its receptors S1P1 and S1P3 and the subsequent activation of the PI3K/Akt and the MEK/ERK pathways (Tao et al., 2010). The central role of mitochondria as targets of HDL-mediated myocyte survival after I/R is confirmed by two evidences. First, the administration of sHDL prevents mitochondrial granulation and disorganization in isolated hearts undergoing I/R (Marchesi et al., 2008). Second, apoA-I-deficient mice display impaired mitochondrial function resulting in higher infarct size after LAD compared to wild-type mice. Interestingly, the administration of coenzyme Q to apoA-I null mice normalizes electron transfer rate from complex II to complex III of the electron transfer chain and reduces infarct size (Dadabayev et al., 2014).

### Endothelial Cells

The HDL-mediated increase of prostanoids and nitric oxide levels after I/R suggests that HDL-mediated cardioprotection is due at least in part to their role in promoting vasodilation. In addition, it is well known that both HDL and sHDL can inhibit the expression of CAMs, which favor circulating cell adhesion to the endothelial layer (Barter et al., 2002; Calabresi et al., 2003a); indeed, HDL can limit the expression of CAMs, neutrophil binding and transmigration in activated endothelial cells and in several *in vivo* models of I/R or acute inflammation (Cockerill et al., 2001a,b; Paterno et al., 2004; Theilmeier et al., 2006).

Interestingly, HDL increase hypoxia-mediated angiogenesis in coronary artery endothelial cells; HDL promote endothelial cell migration, proliferation, and tubulogenesis by acting on the post-translational regulation of the hypoxia-inducible factor-1/vascular endothelial growth factor axis, with a mechanism requiring HDL interaction with the scavenger receptor SR-BI and the activation of PI3K pathway (Prosser et al., 2014; Tan et al., 2014). The pro-angiogenic effect of HDL can be also mediated by their ability to promote the differentiation of peripheral blood monocytes into endothelial progenitor cells, via activation of the PI3K/Akt/NO pathway (Sumi et al., 2007).

### Infiltrating Cells

An HDL-mediated reduction of neutrophil recruitment in the ischemic tissues was demonstrated *in vivo* in different models of I/R (Cockerill et al., 2001b; Thiemermann et al., 2003; Cuzzocrea et al., 2004; Theilmeier et al., 2006; Lapergue et al., 2010); infiltrating cells may considerably contribute to I/R injury by releasing molecules and enzymes with pro-inflammatory, pro-oxidant, and proteolytic activities. HDL activity on infiltrating cells could be particularly relevant in cerebral ischemia, where the breakdown of the blood brain barrier allows the extravasation of plasma proteins and leukocytes and the development of cerebral edema (Ballabh et al., 2004). Activated leukocytes are thought to take part in this process, since after degranulation they release enzymes responsible for the proteolysis of extracellular matrix (Gidday et al., 2005). A recent elegant study performed on cerebral endothelial cells and leukocytes under oxygen-glucose deprivation conditions showed that the presence of HDL helped in maintaining endothelial layer integrity by inhibiting leukocytes activation (Bao et al., 2013).

## Conclusion

Plasma-derived and sHDL have been clearly shown to exert protective effects against I/R injury in animal models. The protective effects are related to HDL ability to interfere with cellular processes known to play a role in myocardial I/R injury. Interestingly, sHDL are cardioprotective even when administered after the ischemic insult, supporting the therapeutic potential of sHDL infusion to reduce I/R injury. sHDL are currently under clinical development for plaque stabilization/regression in the setting of ACS, primarily because of their ability to rapidly remove cholesterol from the arterial wall (Nissen et al., 2003; Tardif et al., 2007). If administered promptly after ACS, sHDL could also promote acute protection against I/R damage. Based on pre-clinical data summarized in the review, this hypothesis warrants clinical investigation, and could be also extended to other organs, since the pathologic mechanisms targeted by HDL in myocardial I/R are likely not specific to the heart.

## Author Contributions

MG has drafted the work; MG, LC, and GF have critically revised the work and approved the final version to be published.

## Conflict of Interest Statement

The authors declare that the research was conducted in the absence of any commercial or financial relationships that could be construed as a potential conflict of interest.
